# Color recycling: metabolization of apocarotenoid degradation products suggests carbon regeneration via primary metabolic pathways

**DOI:** 10.1007/s00299-022-02831-8

**Published:** 2022-01-22

**Authors:** Julian Koschmieder, Saleh Alseekh, Marzieh Shabani, Raymonde Baltenweck, Veronica G. Maurino, Klaus Palme, Alisdair R. Fernie, Philippe Hugueney, Ralf Welsch

**Affiliations:** 1 Faculty of Biology II, University of Freiburg, 79104 Freiburg, Germany; 2 Max-Planck-Institute for Molecular Plant Physiology, Am Mühlenberg 1, 14476 Potsdam, Germany; 3 Center for Plant Systems Biology and Biotechnology, 4000 Plovdiv, Bulgaria; 4 Department of Plant Production and Genetics, School of Agriculture, Shiraz University, Shiraz, Iran; 5 Université de Strasbourg, INRAE, SVQV UMR-A 1131, 68000 Colmar, France; 6 Department of Molecular Plant Physiology, Institute of Molecular Physiology and Biotechnology of Plants, University of Bonn, Kirschallee 1, 53115 Bonn, Germany

**Keywords:** Apocarotenoids, Carotenoid degradation, Glutathione, Glyoxal, Glyoxalase, Methylglyoxal

## Abstract

**Key message:**

Analysis of carotenoid-accumulating roots revealed that oxidative carotenoid degradation yields glyoxal and methylglyoxal. Our data suggest that these compounds are detoxified via the glyoxalase system and re-enter primary metabolic pathways.

**Abstract:**

Carotenoid levels in plant tissues depend on the relative rates of synthesis and degradation. We recently identified redox enzymes previously known to be involved in the detoxification of fatty acid-derived reactive carbonyl species which were able to convert apocarotenoids into corresponding alcohols and carboxylic acids. However, their subsequent metabolization pathways remain unresolved. Interestingly, we found that carotenoid-accumulating roots have increased levels of glutathione, suggesting apocarotenoid glutathionylation to occur. In vitro and *in planta* investigations did not, however, support the occurrence of non-enzymatic or enzymatic glutathionylation of β-apocarotenoids. An alternative breakdown pathway is the continued oxidative degradation of primary apocarotenoids or their derivatives into the shortest possible oxidation products, namely glyoxal and methylglyoxal, which also accumulated in carotenoid-accumulating roots. In fact, combined transcriptome and metabolome analysis suggest that the high levels of glutathione are most probably required for detoxifying apocarotenoid-derived glyoxal and methylglyoxal via the glyoxalase pathway, yielding glycolate and d-lactate, respectively. Further transcriptome analysis suggested subsequent reactions involving activities associated with photorespiration and the peroxisome-specific glycolate/glyoxylate transporter. Finally, detoxified primary apocarotenoid degradation products might be converted into pyruvate which is possibly re-used for the synthesis of carotenoid biosynthesis precursors. Our findings allow to envision carbon recycling during carotenoid biosynthesis, degradation and re-synthesis which consumes energy, but partially maintains initially fixed carbon via re-introducing reactive carotenoid degradation products into primary metabolic pathways.

**Supplementary Information:**

The online version contains supplementary material available at 10.1007/s00299-022-02831-8.

## Introduction

Carotenoids are tetraterpenes serving a multitude of functions in plants as essential photosynthetic pigments, substrates for phytohormone biosynthesis and colorants of fruits and flowers contributing to sexual reproduction of plants (Yuan et al. 2015; Baranski and Cazzonelli 2016). Carotenoid biosynthesis in plants has become very well established and extensively reviewed in the past decades (Cazzonelli et al. 2009; Wurtzel 2019). In contrast, knowledge on carotenoid catabolism, initiated by oxidative cleavage of their polyunsaturated hydrocarbon backbone, has only improved more recently and is a key determinant of steady-state carotenoid levels. In most tissues, flux through carotenoid biosynthesis is by far higher than the steady-state levels observed, a fact that supports the significance and extent of carotenoid turnover (Simkin et al. 2003; Lätari et al. 2015; Koschmieder et al. 2020). The research focus has long been on enzymatic carotenoid catabolism by carotenoid cleavage dioxygenases (CCDs) and nine-cis-epoxy-carotenoid dioxygenases (NCEDs), initiating biosynthesis of abscisic acid (ABA), strigolactones and other novel bioactive compounds, but also leading to non-specific cleavage yielding apocarotenoids of different chain lengths (Hou et al. 2016; Wang and Bouwmeester 2018; Moreno et al. 2020).

Several lines of evidence have put a spotlight on non-enzymatic oxidative degradation. In chloroplasts, photooxidation was reported to be the predominant carotenoid degradation process, producing apocarotenoids as light stress signals (Simkin et al. 2003; Beisel et al. 2010; Ramel et al. 2012; Lätari et al. 2015). Moreover, knockout of CCD1 and CCD4 genes, previously thought to be key to carotenoid catabolism, barely affects steady-state carotenoid levels (Gonzalez-Jorge et al. 2013; Lätari et al. 2015). Lastly, in vitro and *in planta* investigations suggest that non-enzymatic carotenoid oxidation yields polymeric aggregates, called co-polymers, most likely representing the largest portion of degraded carotenoid in high carotenoid tissues such as carrot roots or Golden Rice endosperm (Britton 1995; Burton et al. 2014; Schaub et al. 2017, 2018; Mogg and Burton 2020). Co-polymers slowly decompose by scission to yield apocarotenoids and many short-chain metabolites upon secondary cleavage of remaining double bonds and further metabolism.

**Fig. 1:**
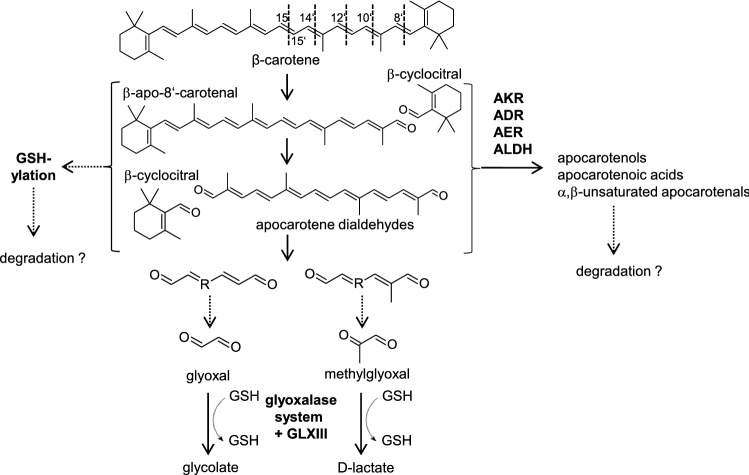
β-Carotene turnover pathways. β-Carotene oxidation primarily yields β-apocarotenoids of various chain lengths (numbers indicate the reacting double bond position), such as the cleavage product pair β-apo-8′-carotenal and β-cyclocitral shown as an example. Linear apocarotenals are modified by different enzymes which generate apocarotenols, apocarotenoic acids and α, β-unsaturated aocarotenals which might be subjected to further degradation. Alternatively, apocarotenoid–GSH adducts might be formed, which might enter known degradation pathways. Secondary and repeated oxidation of apocarotenoids yields linear apocarotene dialdehydes and as potential end products cytotoxic methylglyoxal (C3) and glyoxal (C2). The GSH-dependent glyoxalase pathway represents their predominant detoxification pathway yielding products which can enter primary metabolism. *AKR* aldo–keto reductase, *ADR* aldehyde reductase, *AER* 2-alkenal reductase, *ALDH* aldehyde dehydrogenase. Arrows with solid lines indicate identified pathways, while dotted lines indicate putative pathways

Aiming at investigating this largely unknown metabolism downstream of apocarotenoid formation, we recently analyzed roots of *Arabidopsis* plants which accumulate dramatically increased amounts of β-carotene (Koschmieder et al. 2020). This was achieved by overexpression of the first and rate-limiting enzyme of the carotenoid pathway, phytoene synthase (PSY), which is frequently applied also in biotechnological applications to increase the provitamin A content (Paine et al. 2005; Welsch et al. 2010; Bai et al. 2016; Diepenbrock et al. 2020). Using carotenoid-accumulating *Arabidopsis* roots, we found that β-apocarotenoids which represent reactive electrophile species (RES) with α,β-unsaturated carbonyl moieties are metabolized by a set of enzymes so far known as detoxifiers of reactive carbonyl species (RCS; Fig. 1). These enzymes convert aldehydes/ketones into less reactive and less toxic alcohols, carboxylic acids or a,β-unsaturated aldehydes. Namely, these are aldehyde dehydrogenases ALDH3H1 and ALDH3I1, aldo–keto reductases AKR4C8 and AKR4C9 and the 2-alkenal reductase AER (Mano et al. 2005, 2019; Yamauchi et al. 2012; Mano 2012). However, mechanisms responsible for subsequent metabolization of apocarotenoids or apocarotenoids modified by the enzymes identified remain unexplored.

Glutathionylation of apocarotenoids at the α,β-unsaturated double bond by glutathione (GSH) S-transferases (GSTs) might represent an effective detoxification mechanism, which would tag apocarotenoids for further degradation as known for other compounds, e.g., oxilipins (Davoine et al. 2005, 2006). Interestingly, 11 out of 23 *Arabidopsis* GSTs of the tau subfamily (GSTU) are reported to mediate glutathionylation of RCS such as acrolein or 4-hydroxy-(E)-2-nonenal in vitro (Mano et al. 2017). However, the substrate specificity of most plant GSTs remains unknown. Additionally, non-enzymatic glutathionylation of many compounds such as oxylipins and alkenes occurs even at physiological pH without catalysts in vitro (Esterbauer et al. 1975). Considering the above similarities between RCS and apocarotenoid metabolism and considering that β-apocarotenoids as well as many RCS are lipophilic hydrocarbons, the formation of apocarotenoid–GSH adducts appears plausible, but there is scarce experimental evidence for this. For instance, non-enzymatic glutathionylation of the C10 apocarotenoid citral in vitro was reported (Esterbauer et al. 1975) and a specific GST was shown to be capable of binding zeaxanthin in human macula (Bhosale et al. 2004).

An alternative degradation mechanism for (apo)carotenoids is their oxidative cleavage into very short-chain compounds. We reported that transgenic *Arabidopsis* calli and roots accumulating β-carotene and β-apocarotenoids also accumulate the reactive carbonyl species methylglyoxal (C3) and glyoxal (C2) (Schaub et al. 2018; Koschmieder et al. 2020), potentially representing terminal oxidation products of β-carotene and/or carotenoids in general (Fig. 1). These products have been confirmed as oxidation products of β-carotene in vitro under ozonolysis (Benevides et al. 2011). Upon oxygen exposure of synthetic β-carotene, many compounds including methylglyoxal, glyoxal and the central metabolites pyruvic acid and succinic acid were reported to appear, their occurrence coinciding with β-carotene co-polymerization (Mogg and Burton 2020). However, methylglyoxal and glyoxal are ubiquitous, highly cytotoxic compounds produced both enzymatically and non-enzymatically from several pathways which complicates their traceability from an apocarotenoid origin (Yadav et al. 2008; Hoque et al. 2016). Methylglyoxal and glyoxal are mainly detoxified via the GSH-dependent glyoxalase pathway yielding D-lactate and glycolate which are further fueled into the tricarboxylic acid and the Calvin–Benson cycle via the photorespiratory cycle, respectively (Thornalley 1990; Maurino and Engqvist 2015; Dellero et al. 2016; Welchen et al. 2016; Modde et al. 2016; Schmitz et al. 2017).

In this work, we aimed at further investigating the largely unknown carotenoid metabolism downstream of apocarotenoid formation, based on our previously reported model system of transgenic *Arabidopsis* roots which over-accumulate β-carotene and β-apocarotenoids (Maass et al. 2009; Álvarez et al. 2016; Koschmieder et al. 2020). We conducted comprehensive in vitro investigations as well as mass spectrometry-based metabolite analysis and transcriptome analysis in transgenic *Arabidopsis* roots to address the possible occurrence of non-enzymatic and GST-mediated enzymatic glutathionylation of β-apocarotenoids. A combination of different experimental approaches was used to further investigate the origin and fate of methylglyoxal and glyoxal from carotenoid degradation *in planta* and also the role of GSH in their detoxification. Our results suggest continuous breakdown and partial recycling of carotenoid-derived carbon via primary metabolic pathways and unravel a hitherto unrecognized function of enzymes predominantly associated with photorespiration in heterologous tissues.

## Results

### Glutathione levels increase in response to β-carotene and β-apocarotenoid accumulation

We recently reported the conversion of β-apocarotenoids by a set of enzymes so far known as detoxifiers of reactive carbonyl species (RCS), converting aldehydes/ketones into less reactive and potentially toxic alcohols, carboxylic acids or α,β-saturated aldehydes (Fig. 1, Koschmieder et al. 2020). Considering that (non-)enzymatic glutathionylation is an additional, potentially more effective detoxification pathway for RCS (Mano et al. 2019), we investigated the role of (non-)enzymatic glutathionylation in apocarotenoid metabolism.

**Fig. 2:**
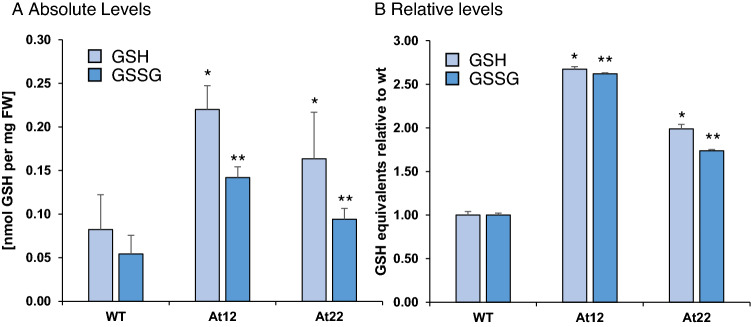
GSH and GSSG content in *Arabidopsis* roots. GSH and GSSG levels were determined by colorimetric DTNB assays in roots of *Arabidopsis* wild type (WT) and two lines with increased carotenoid pathway activity achieved through *AtPSY* overexpression (*At12*, *At22*). **A** Absolute levels of GSH and GSSG, **B** relative levels of GSH and GSSG, each normalized to the levels in wild-type roots. Results are mean ± SD from at least three biological replicates. Significant difference relative to the wild type determined by Student’s *t* test, with *p < 0.05, **p < 0.01

Cellular levels of GSH are maintained by GSH reductase catalyzing the reduction of oxidized GSH, which forms the dimer glutathione disulfide (GSSG) and thus regenerates GSH. First, cellular levels of reduced and oxidized glutathione (GSH and GSSG, respectively) in roots of two independent *PSY*-overexpressing, apocarotenoid-accumulating *Arabidopsis* lines (*At12* and *At22*) were determined. Interestingly, both lines had significantly increased levels of GSH and GSSG by a factor of 1.8 and 2.5, respectively, with the reduced and oxidized form increasing to a practically identical degree (Fig. 2A, B). Thus, the ratio of GSH and GSSG, a measure for the cell redox state, remained unchanged. The analysis of our recently obtained RNA-Seq data (Koschmieder et al. 2020) suggests that increased GSH accumulation is not due to transcriptional induction of the rate-limiting enzyme of GSH biosynthesis, glutamyl-cysteine ligase (At4g23100), or GSH synthase (At5g27380), with their transcript levels remaining unchanged. It must therefore rather be due to more complex regulatory processes in the metabolism of GSH, the supply of its precursor L-cysteine, which has also been considered rate limiting, or the interplay with the ascorbic acid–GSH cycle (Richman and Meister 1975; Hasanuzzaman et al. 2017). In conclusion, GSH increases in response to increased β-carotene and β-apocarotenoid accumulation and might allow sufficient detoxification of reactive compounds formed in subsequent catabolic processes.

### Non-enzymatic glutathionylation does not contribute to β-carotene metabolism

Non-enzymatic glutathionylation is known to detoxify many compounds such as apocarotenoid citral, hydrophobic oxylipins and alkenes. This reaction even occurs at physiological pH in phosphate buffer and without catalysts in vitro and corresponding GSH adducts are often detectable *in planta* (Esterbauer et al. 1975; Davoine et al. 2005, 2006). We therefore investigated non-enzymatic glutathionylation of β-apocarotenoids ranging from C30 to C10 in vitro. High molar excess of GSH is known to strongly favor glutathionylation of reactive electrophiles (Esterbauer et al. 1975). Therefore, assays were conducted with GSH in up to 150-fold molar excess and at near-physiological concentrations of 10 mM for several hours in both phosphate buffer with neutral pH of 7.4 and Tris buffer with an alkaline pH of 9.0. We analyzed for possible reductions of the initial amounts of apocarotenoids as indicator for apocarotenoid–GSH adduct formation. However, the incubations did not reveal a decrease of free apocarotenoid levels at either pH in vitro (Table 1). In contrast, 4-hydroxynonenal, a fatty acid degradation product known to be detoxified by non-enzymatic and enzymatic glutathionylation (Esterbauer et al. 1975; Mano et al. 2019), was readily consumed at pH 7.4 and equimolarity with GSH within only 15 min (Table 1). Accordingly, there was no indication of β-apocarotenoids readily undergoing glutathionylation in vitro in the absence of catalysts.

**Table 1:** Non-enzymatic glutathionylation of β-apocarotenoids

Substrate	Initial substrate (µM)	Initial GSH (mM)	% Remaining substrate
β-8-apo	60	10	102
β-10-apo	60	10	85
β-12-apo	60	5	98
β-14-apo	60	5	106
β-13-apo	60	5	104
β-15-apo	60	10	99
β-11-apo	60	10	97
β-8,8′-di-apo	60	5	97
3OH-β-12-apo	60	5	104
β-ionone	60	10	92
β-cyclocitral	60	10	100
4-HNE	120	120	25

Remaining percentage of free, non-glutathionylated apocarotenoid after 2.5 h of incubation with high molar excess of GSH at pH 9.0, determined by HPLC. The remaining percentage of free, non-glutathionylated 4-hydroxynonenal (4-HNE) as positive control for non-enzymatic glutathionylation was also determined by HPLC, after incubation for 15 min at equimolar GSH concentration and pH 7.4. Results are representative single measurements

### Investigation on enzymatic apocarotenoid glutathionylation by GSTs

**Fig. 3:**
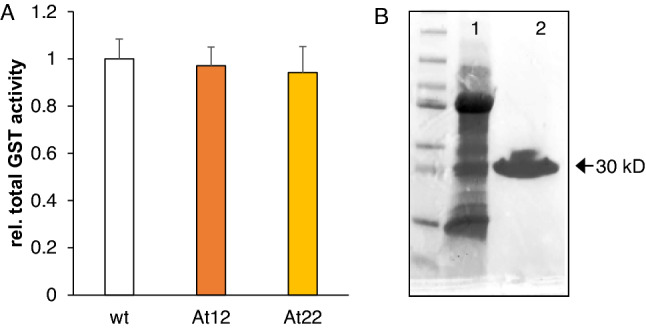
Total GST activity in *Arabidopsis* roots and purification of endogenous GSTs from *Arabidopsis* leaves. **A** Relative GST activity in roots of *Arabidopsis* wild type (wt) and two lines with increased carotenoid pathway activity achieved through *AtPSY* overexpression (*At12*, *At22*), determined by colorimetric CDNB assays. Results are mean ± SD from at least three biological replicates. No significant difference relative to the wild type was determined (Student’s *t* test, *p* < 0.05). **B** Endogenous GSTs were purified from *Arabidopsis* leaves using GSH sepharose 4B. GST purification was monitored by 4–20% SDS-PAGE. Lane 1 represents 60 µg of total soluble *Arabidopsis* leaf protein, lane 2 represents 3.5 µg of purified protein, with a slightly diffuse but single dominant band at appr. 30 kD, the expected size for *Arabidopsis* GSTs. GST activity of the purified protein fraction was determined using a colorimetric DTNB assay

Next, we investigated whether apocarotenoid glutathionylation requires enzymatic catalysis by GSTs. *Arabidopsis* GSTs of the tau subfamily (GSTU) have recently been suggested to mediate detoxification of RCS by glutathionylation (Yamauchi et al. 2012; Mano et al. 2019), with the substrate specificity of many GSTUs still being unknown. Transcriptome analysis of *PSY*-overexpressing *Arabidopsis* roots revealed that ten GSTs were induced upon carotenoid accumulation, mostly by a factor of 3 and included GSTUs and GSTFs of mostly unknown substrate specificity (Supplemental Table S1). We determined the total GST activity in *Arabidopsis* roots using a colorimetric GST activity assay with the universal GST substrate 1-chloro-2,4-dinitrobenzene (CDNB). We found that the total GST activity is not increased (Fig. 3A).

To exclude that an only weak, specific apocarotenoid glutathionylation by GSTs activity is suppressed by interfering compounds in the plant extract, we decided to refine the analysis with a protein fraction enriched with GST enzymes. Given the multitude of *Arabidopsis* GSTs (Dixon and Edwards 2010), we did not aim at heterologous expression and in vitro testing for single GSTs, but decided to non-selectively co-purify endogenous GSTs of the tau and phi subfamilies from *Arabidopsis* leaves using immobilized GSH affinity chromatography (Edwards and Dixon 2005). The method allowed us to obtain approximately 250 µg of highly pure GSTs from about 100 g of *Arabidopsis* leaves (Fig. 3B) which showed a high specific activity to glutathionylate the control substrate CDNB with 5.3 nmol CDNB min^−1^ µg^−1^ in vitro. However, as indicated by unchanged substrate amounts after several hours of incubation at physiological pH of 7.4 with equimolar amounts of GSH (Table 2), in contrast to the control substrate, apocarotenoids (C30–C10) were not glutahionylated. Currently, we cannot exclude that our assay was not sufficiently sensitive to detect a weak apocarotenoid glutathionylation activity, as only the transcription of seven of the AtGSTs is upregulated in *At12* and *At22* (Supplemental Table S1). This remains to be explored using recombinant enzymes in future investigations.

**Table 2:** Enzymatic glutathionylation of β-apocarotenoids with total GST preparation from *Arabidopsis* leaves

Substrate	Remaining substrate
β-8-apo	95
β-10-apo	107
β-12-apo	106
β-14-apo	100
β-15-apo	99
β-13-apo	100
β-11-apo	96
β-8,8′-di-apo	100
3OH-β-12-apo	103
β-ionone	99
β-cyclocitral	101

Remaining percentage of free, non-glutathionylated apocarotenoid after 4 h of incubation with equimolar concentration of GSH (120 µM apocarotenoid and 120 µM GSH) at pH 7.4, determined by HPLC. Results are representative single measurements

Lastly, we investigated whether GSH adducts of apocarotenoids could be detected in *At12* and *At22* roots by comprehensive metabolite extraction followed by LC–MS analysis. Molecular ions corresponding to putative adducts of the apocarotenoids detected in the *At12* and *At22* lines (Koschmieder et al. 2020) were searched based on their predicted molecular formula, together with other GSH adducts described previously (Davoine et al. 2005). Besides OPDA-GSH, putative GSH adducts of β-apo-10-carotenoic acid, β-apo-11-carotenoic acid and β-apo-12-carotenol could be detected in all root extracts (Supplemental Figure S1). However, these putative adducts did not accumulate significantly more in the *At12* and *At22* lines than in the wild type, despite the high levels of apocarotenoid accumulation in the transgenic lines (Koschmieder et al. 2020).

### Methylglyoxal and glyoxal as terminal oxidation products of β-carotene

Our experimental data show that GSH and GSSG levels rise in response to β-carotene and β-apocarotenoid accumulation. However, despite increased availability of GSH for potential detoxification processes related to carotenoid metabolism, we could not find any experimental evidence in vitro and *in planta* supporting non-enzymatic or enzymatic glutathionylation by GSTs for β-apocarotenoids ranging from C10 to C30.

We recently showed that *Arabidopsis* calli and roots overexpressing *PSY* and accumulating β-carotene and β-apocarotenoids also accumulate methylglyoxal and glyoxal (Schaub et al. 2018; Koschmieder et al. 2020), two reactive carbonyl species identified also as in vitro degradation products of β-carotene (Benevides et al. 2011; Mogg and Burton 2020). Direct investigations on whether these metabolites also originate from carotenoids *in planta* are hampered by the difficulties arising from cellular uptake of lipophilic (apo)carotenoids into plastids and/or the multitude of pathways sharing IPP as common precursor. Thus, traditional pulse-chase experiments as conceived to uncover, e.g., the plastid-localized MEP pathway, cannot be performed (Lichtenthaler et al. 1997). If not originating from apocarotenoid oxidation, a possible source of methylglyoxal and even more so of glyoxal is considered to be lipid peroxidation (Paudel et al. 2016). Lipid peroxidation is reported to occur also at high carotenoid levels and high oxygen partial pressure (Yanishlieva et al. 1998; McNulty et al. 2007). However, we previously reported that roots of *Arabidopsis* lines accumulating β-carotene and β-apocarotenoids do not accumulate lipid peroxidation intermediates or upregulate lipid stress-related genes (Koschmieder et al. 2020). It is therefore reasonable to conclude that accumulating methyglyoxal and glyoxal in *At12* and *At22* rather originate from continuous carotenoid and apocarotenoid turnover. One known degradation mechanism for the ionone rings of carotenoids is their oxidative cleavage into geronic acid (Burton et al. 2016; Schaub et al. 2017). The remaining linear carbohydrate backbone can theoretically be fully oxidized into methylglyoxal and glyoxal. Importantly, considering a complete degradation of the β-carotene backbone, equimolar amounts of both metabolites are generated.

### Involvement of glyoxalase activities

In plants, methylglyoxal and glyoxal detoxification and metabolism strongly depend on cellular levels of reduced GSH and the GSH-dependent glyoxalase (GLX) pathway (Yadav et al. 2005b, a; Upadhyaya et al. 2011; Schmitz et al. 2017). In agreement, we observed increased cellular levels of reduced GSH in *At12* and *At22* roots, accumulating both methylglyoxal and glyoxal requiring detoxification (Fig. 2). In the glyoxalase pathway, glyoxalase I (GLXI) converts methylglyoxal and GSH to S-D-lactoylglutathione which is converted by glyoxalase II (GLXII) to D-lactate, liberating GSH. Glyoxalase III (GLXIII) enzymes directly convert methylglyoxal to D-lactate in a single step, without using GSH or any other cofactor. Moreover, D-lactate dehydrogenase (D-LDH) is also associated with the glyoxalase system and metabolizes D-lactate into pyruvate which enters into the TCA cycle (Engqvist et al. 2015; Welchen et al. 2016).

Based on previous findings, glyoxalase GLXI activity is rate-limiting for the entire glyoxalase pathway and directly proportional to the GSH concentration available to the enzyme (Lages et al. 2012; Rabbani and Thornalley 2014; Nigro et al. 2017). In view of this, our findings suggest that increased GSH levels in *At12* and *At22* roots probably increase glyoxalase pathway activity and flux, allowing for increased detoxification of apocarotenoid-derived methyglyoxal and glyoxal (Fig. 1).

Additionally, we investigated upregulation of the glyoxalase GLX pathway and GLX-like at the transcript and protein level. Interestingly, the expression of 2 of the 11 known GLXI and GLXI-like genes in *Arabidopsis* (Schmitz et al. 2017, 2018) were upregulated in *At12* and *At22*; among the strongest GLXI-like;7 (At1g80160) by factor 29 and 10, respectively, and GLXI-like;4 (At1g15380) by factor 4 and 3, respectively (Table 3). Notably, however, GLXI-like;4 and GLXI-like;7 are considered non-functional glyoxalases, although a final confirmation with recombinant proteins is still required (Schmitz et al. 2018). Genes annotated as GLXII were not found to be differentially regulated in *PSY*-overexpressing roots. Interestingly, the expression of GLXIII (At3g02720) encoding an enzyme with highest in vitro activity on methylglyoxal and glyoxal among all GLXIII enzymes (Kwon et al. 2013) was not upregulated, while the expression of GLXIII (At3g54600), encoding an enzyme of low in vitro activity on methylglyoxal, was upregulated by factor 2 and 4 in A12 and At22, respectively (Table 3).

**Table 3:** DEGs in carotenoid-accumulating *Arabidopsis* roots

Locus ID	Name	Description	Fold change	
			*At22*	*At12*
Glyoxylase cycle				
At3g02720	GLXIII	Glyoxylase III	n.s	n.s
At3g54600	GLXIII	Glyoxylase III	4	2
Tricarboxylic acid cycle				
At5g03860	MLS	Malate synthase	13.5	13.4
At5g09660	MDH	Malate dehydrogenase	3.6	3.1
Photorespiration				
At1g32080	PLGG1	Glycolate/glycerate translocator 1	11	5
At3g14420	GOX1	Glycolate oxidase	5	4
At3g14415	GOX2	Glycolate oxidase	n.s	n.s
At2g13360	SGAT	Serine:glutamate aminotransferase	4	8
Glycolysis				
At1g43670	FBP	Fructose-1,6-bisphosphatase	2.2	2.2
At4g26520	FBA7	Fructose-bisphosphate aldolase	n.s	3.2
At3g26650	GAPA1	Glycerinaldehyde-3-phosphate dehydrogenase	n.s	2.0
At1g12900	GAPA2	Glycerinaldehyde-3-phosphate dehydrogenase	5.3	3.9
At5g62840		Phosphoglycerate mutase	2.3	2.6
At1g09932		Phosphoglycerate mutase	n.s	3.8
At3g60420		Phosphoglycerate mutase	5.5	7.2
Other Pathways				
At5g65690	PCK2	Phosphoenolpyruvate carboxykinase 2	5.9	6.7
At4g37870	PCK1	Phosphoenolpyruvate carboxykinase	2.6	2.6
At1g80160	GLXI-like;7	Glyoxalase-like	10	29
At1g15380	GLXI-like;4	Glyoxalase-like	3	4

RNA-Seq-determined expression changes in roots of *PSY*-overexpressing lines *At22* and *At12* relative to wild-type roots

**Fig. 4:**
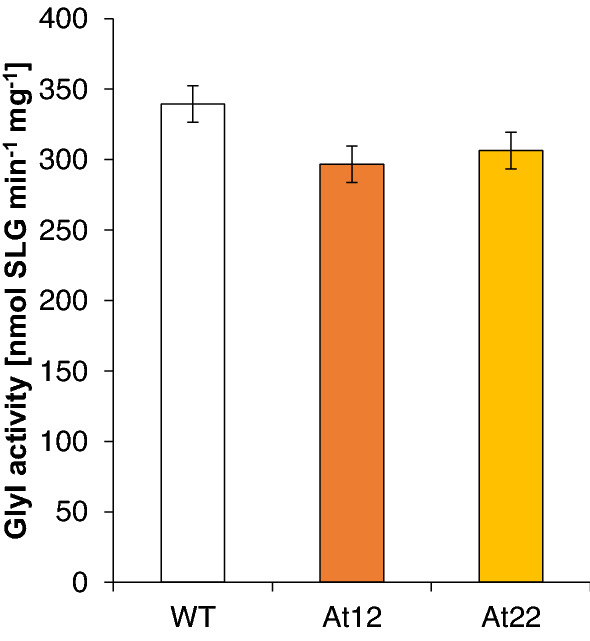
Total GLXI activity in *Arabidopsis* roots. Total activity of GLXI enzymes in roots of *Arabidopsis* wild type (WT) and two lines with increased carotenoid pathway activity achieved through *AtPSY* overexpression (*At12*, *At22*) were determined photometrically. Results are mean ± SD from at least three biological replicates. No significant difference relative to the wild type was determined (Student’s *t* test, *p* < 0.05). *SLG* S-lactoylglutathione

Therefore, transcriptome analysis in *AtPSY*-expressing roots revealed no evidence for transcriptional induction of the canonical glyoxalase GLX pathway, rather pointing toward induction of GLXIII for one-step conversion of the cytotoxic metabolites. In line with this, we found that overall activity of GLXI, the rate-limiting enzyme of the pathway in plants and in mammals (Rabbani and Thornalley 2014), remained unchanged in *A12* and *At22* roots (Fig. 4). We propose that in addition to GLXIII-mediated methylglyoxal detoxification, increased availability of both substrates—GSH and apocarotenoid-derived methyglyoxal and glyoxal in *A12* and *At22* roots—is likely sufficient to increase flux through the glyoxalase system without the need for induction of the rate-limiting enzyme GLXI at the transcript or enzyme level.

### Metabolomic analysis indicates methylglyoxal and glyoxal detoxification

**Fig. 5:**
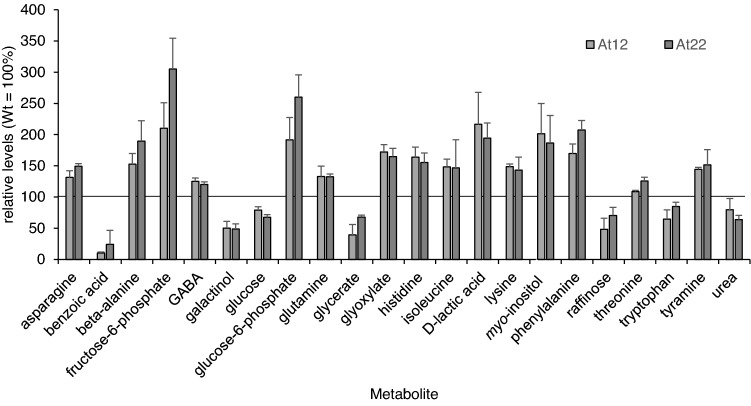
Metabolite analysis of carotenoid-accumulating *Arabidopsis* roots. Metabolites were analyzed from three biological replicates of roots from hydroponically grown wild-type and *AtPSY*-overexpressing *Arabidopsis* lines (Supplemental Figure S2). Metabolites with significant differential accumulation (*p* ≤ 0.05, Student’s *t* test) compared to wild-type roots in both *AtPSY*-overexpressing lines are represented relative to the amounts detected in the wild type

Given the indications for an enhanced detoxification of apocarotenoid-derived methylglyoxal and glyoxal, we decided to perform a metabolomic analysis in roots of hydroponically grown plants. Relative amounts of a set of 71 metabolites were quantified in wild-type and transgenic lines by GC–MS after derivatization (Lisec et al. 2006) and differential metabolite accumulation among lines was tested (Supplementary Figure S2). Among the metabolites analyzed, we focused on those which were significantly different from wild-type roots grown in parallel and responded consensually in both carotenoid-accumulating lines, thus either increasing or decreasing metabolite levels compared with wild-type roots (Fig. 5).

Interestingly, we found evidence for the detoxification of both apocarotenoid-derived breakdown products glyoxal and methylglyoxal. Firstly, D-lactate levels increased by twofold in both carotenoid-accumulating lines compared to wild-type levels, suggesting its origin from apocarotenoid-derived methylglyoxal. Although mitochondrial D-LDH was not induced transcriptionally in neither *At12* nor *At22*, we suggest that the capacity is sufficient to convert D-lactate into pyruvate, also considering that other metabolic pathways to detoxify D-lactate are not known in plants (Maurino and Engqvist 2015; Welchen et al. 2016). Levels of pyruvate were similarly unchanged, however, especially assuming an increased demand for plastid-derived IPP for carotenoid biosynthesis in *PSY*-overexpressing roots. We consider that methylglyoxal-derived pyruvate might readily be used for IPP biosynthesis via the MEP pathway, thus explaining its constant amounts like in wild-type roots.

**Fig. 6:**
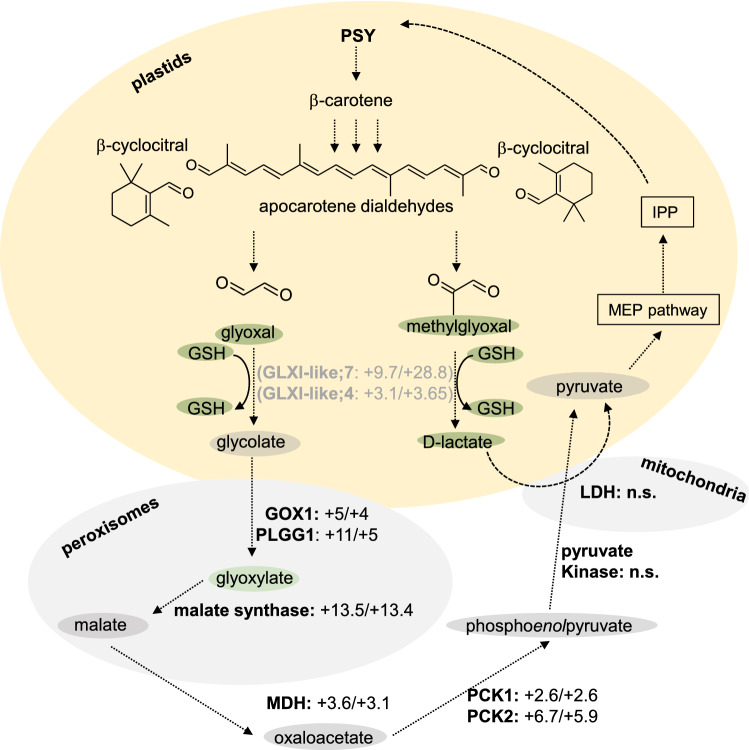
Metabolite and transcript changes in carotenoid-accumulating roots. Essential metabolites for the putative apocarotenoid metabolization pathways are included. Data are based on transcriptome and metabolome comparisons of two carotenoid-accumulating lines *At12* and *At22* against wild-type roots. Metabolites found to be unchanged are marked with gray background, metabolites with increased abundance in carotenoid-accumulating roots are marked with green background. Enzymes catalyzing essential metabolite conversions are indicated, expression level differences in *At12*/*At22* versus wild-type roots are given; n.s. indicates that no significant transcript changes were determined in RNA-Seq analysis. Dashed lines indicate that several enzymes are involved. *GLXI-like* glyoxalaseI-like, *GOX* glycolate oxidase, *GSH* glutathione, *IPP* isopentenyl diphosphate, *LDH* lactate dehydrogenase, *MEP* methylerythritol phosphate, *MDH* malate dehydrogenase, *PLGG* plastidal glycolate/glycerate translocator, *PCK* phosphoenolpyruvate carboxykinase, *PSY* phytoene synthase

In addition to D-lactate which is the conversion product of methylglyoxal, we found glyoxylate which is likely derived from glyoxal to be increased by twofold in apocarotenoid-accumulating lines. The formation of glyoxylate from glyoxal involves the intermediate glycolate which was unchanged in apocarotenoid-accumulating roots and requires the activities of glycolate oxidase (GOX; converting glycolate to glyoxylate) and the glycolate transporter PLGG1 (At1g32080; Fig. 6). These two proteins are involved in photorespiration in photosynthetically active tissues (Nunes-Nesi et al. 2014; Maurino and Engqvist 2015; Modde et al. 2016). However, photorespiratory enzymes are assumed to fulfill other metabolic requirements in heterotrophic tissues (Engqvist et al. 2015; Schmitz et al. 2020), such as roots which are investigated in this work. As a confirmation, we found transcriptional upregulation of some genes encoding enzymes involved in photorespiration, although transcript levels in roots are very low compared to green tissues (Table 3). Remarkably, the plastidal glycolate/glycerate translocator 1 PLGG1, which exports glycolate from plastids into peroxisomes (Pick et al. 2013), was induced 11-fold. Moreover, the peroxisomal enzymes glycolate oxidase GOX1 (At3g14420), converting glycolate into glyoxylate, and serine:glutamate aminotransferase SGAT (At2g13360) were induced four- and eightfold, respectively (Table 3). Genes encoding proteins involved in photorespiration, namely GOX2 (At3g14415), GDC-T (At1g11860), GDC-P (At4g33010), SHM1 (At4g37930), HPR1 (At1g68010) and GLYK (At1g80380), were not considerably induced. These findings agree with recent reports on alternative functions of enzymes linked with photorespiration in heterotrophic tissues (Nunes-Nesi et al. 2014; Engqvist et al. 2015; Schmitz et al. 2020). In summary, genes coding for enzymes involved in the steps involving glycolate/glyoxylate metabolism were considerably upregulated. This might represent an alternative biological function of those activities, here in non-photosynthetic tissues, allowing for increased metabolism of apocarotenoid-derived glyoxal ultimately allowing for carbon recycling via primary pathways (Fig. 6).

Similarly, the level of malate which is the product of glyoxylate metabolization catalyzed by malate synthase remained uchanged, while the transcript levels of malate synthase were strongly increased 13-fold in *At12* and *At22*. Likewise, malate dehydrogenase, which converts malate into oxaloacetate, was induced by threefold. Interestingly, and in support of the involvement of malate entry of glyoxal-derived carbon, transcripts of all other enzymes of the tricarboxylic acid cycle (TCA) remained unchanged. Oxaloacetate might further be converted into phosphoenolpyruvate, which is supported by increased expression levels of the enzymes phosphoenolpyruvate carboxykinase and phosphoenolpyruvate carboxykinase 2 (three- and sixfold increases in both lines). Finally, phosphoenolpyruvate can be converted into pyruvate by pyruvate kinase; however, the expression levels of the corresponding enzyme remained unchanged.

### Other metabolites induced in apocarotenoid-accumulating roots

Remarkably, apart from the metabolites of primary carbon metabolic pathways, the levels of a number of amino acids were found to be changed in response to apocarotenoid accumulation. Serine and glycine, substrate and product of the induced photorespiratory SGAT enzyme, respectively, were unchanged, while other amino acids like β-alanine, asparagine, glutamine, lysin, phenylalanine and threonine increased up to twofold in abundance. Interestingly, the same amino acids were increased in fruits of tomato plants with increased carotenoid levels upon overexpression of the fruit-specific phytoene synthase (Fraser et al. 2007). Although tomatoes accumulate mainly linear lycopene and not cyclic β-carotene like the *Arabidopsis* roots investigated in this work, oxidative breakdown of the common carotenoid backbone most likely occurs with similar end products methylglyoxal and glyoxal. This might explain similar responses of primary pathway metabolites including amino acids. Possibly, a number of amino acids can be formed by interconversions mediated by amino transferases. The similarities between lycopene and β-carotene-induced alterations in amino acid abundances suggest similar balancing metabolic responses toward altered primary carbon metabolism which requires further investigations.

Finally, several intermediates of the glycolytic pathway were present in higher abundance, such as fructose-6-P and glucose-6-P increased up to threefold in apocarotenoid-accumulating roots, while glucose levels were slightly reduced. In agreement with this, several enzymes of the pathway were induced: fructose-1,6-bisphosphatase (twofold), aldolase (threefold), glycerinaldehyde-3-phosphate dehydrogenases (fourfold and twofold, respectively) and phosphoglycerate mutases (up to sevenfold; Table 3). The reduced levels of glucose in carotenoid-accumulating roots and the induction of enzymes, indicative of glycolysis (Giegé et al. 2003), suggests that these processes are required to mobilize carbon from glucose to fuel the high carotenoid biosynthesis activity introduced by the transgenic expression of the rate-limiting enzyme PSY.

## Discussion

### Glutathionylation is unlikely to contribute to apocarotenoid metabolism

Non-enzymatic carotenoid turnover generates primary cleavage products, apocarotenoids of various chain lengths, which are subjected to further metabolization by a series of different enzymes. We recently identified that this is mediated by redox enzymes (ALDHs, AKRs, AER, ADR) so far only considered as detoxifiers of reactive carbonyl species (RCS), fatty acid degradation products with α,β-unsaturated double bonds and aldehyde/ketone functionalities (Koschmieder et al. 2020). In addition to enzymatic conversion into less reactive metabolites, the formation of GSH adducts by enzymatic and non-enzymatic processes represents an additional, potentially more effective detoxification pathway known for RCS (Mano et al. 2019). As we determined higher levels of GSH/GSSG in apocarotenoid-accumulating roots, this possibility was taken into account.

However, in vitro, we did not find any indications for the non-enzymatic formation of apocarotenoid adducts, not even upon prolonged incubation in the presence of high molar excess of GSH and at alkaline pH conditions which are known to both strongly favor glutathionylation (Esterbauer et al. 1975). In contrast, the RCS compound 4-HNE was readily glutathionylated even at neutral pH and within minutes, as expected (Esterbauer et al. 1975; Davoine et al. 2005, 2006). Notably, Esterbauer et al. reported the identification of GSH adducts of C10 apocarotenoid citral, although with a very low efficiency, determining 50% glutathionylated citral after 3.5 days of incubation (Esterbauer et al. 1975). In contrast to citral, medium- to long-chained β-apocarotenoids exhibit a conjugated double bond system with delocalized electron density which reduces the polarization of the α,β-unsaturated double bond by electron-withdrawing properties of the aldehyde/ketone which is key to glutathionylation. Moreover, methylation of the α,β-unsaturated double bond as in most β-apocarotenoids strongly reduces glutathionylation (Fig. 1). Therefore, it appears plausible that reactivity towards GSH is strongly reduced or absent for longer-chain, polyunsaturated β-apocarotenoids. Notably, glutathionylation was also not observed for the 3-OH-β-12-apo-carotenal (C25; Table 1). Thus, hydroxylation of the ionone moiety, as present in xanthophyll-derived apocarotenoids, does not increase reactivity towards GSH in vitro.

Increased reactivity of apocarotenoid substrates might be achieved by polarization of the α,β-unsaturated double due to interactions with GST in enzyme-mediated glutathionylations. Several GSTs were upregulated upon accumulation of β-carotene and β-apocarotenoids (Supplemental Table S1), which might indicate their contribution to apocarotenoid gluathionylation. However, a highly active GST fraction affinity-purified from *Arabidopsis* leaves did not show any glutathionylation activity on apocarotenoids (Table 2). We currently cannot exclude that the fraction of apocarotenoid-specific GSTs among the purified GSTs was insufficiently abundant to achieve measurable apocarotenoid glutathionylation. Thus, identification of specific GSTs might require heterologously expressed, recombinant GSTs focusing on those which are upregulated in *At12* and *At22*.

Finally, using a mass spectrometry approach, we could detect similar amounts of only three putative apocarotenoid–GSH adducts in both wild-type and transgenic roots. Thus, in conclusion, our data strongly support that primary apocarotenoids are not subjected to considerable glutathionylation in apocarotenoid-accumulating roots. This corroborates continued oxidation of primary non-enzymatic cleavage products into smaller compounds. Likewise, we conclude that increased GSH/GSSG content in apocarotenoid-accumulating roots is not related to increased GSH demands for apocarotenoid glutathionylation.

### Experimental evidence for metabolization of apocarotenoids into (methyl)glyoxal

Continued oxidative truncation of primary apocarotenoids forms the shortest products methylglyoxal and glyoxal which in fact are present in increased abundance in *Arabidopsis* calli and roots accumulating β-carotene and β-apocarotenoids (Schaub et al. 2018; Koschmieder et al. 2020). Both RCS have also been identified as in vitro degradation products of β-carotene under exposure to oxygen or ozone (Benevides et al. 2011; Mogg and Burton 2020). The main sources of methylglyoxal and glyoxal *in planta* have so far been considered sugar autoxidation, lipid peroxidation and, for the former of the two, especially glycolysis (Schümperli et al. 2007; Paudel et al. 2016; Kammerscheit et al. 2020). However, biochemical and transcriptional evidence excluded lipid peroxidation, a known major source of methylglyoxal and glyoxal, as the cause for the observed accumulation of both RCS in apocarotenoid-accumulating *Arabidopsis* roots (Yanishlieva et al. 1998; Koschmieder et al. 2020). We therefore suggest that methylglyoxal and glyoxal originate from continued carotenoid oxidation.

### Increased GSH levels likely increases flux through the glyoxalase pathway

The glyoxalase pathway dominates detoxification of methylglyoxal and glyoxal using GSH as catalyst (Fig. 1). It comprises the subsequent action of GLXI and GLXII to yield D-lactate from methylglyoxal and glycolate from glyoxal. The sole action of GLXIII can also metabolize the RCS (Schmitz et al. 2017). For instance, in mammalian cells, 99% of both RCS are thought to be detoxified by the glyoxalase pathway (Rabbani and Thornalley 2014). In plants, stress conditions can deplete free GSH which is partially due to its utilization in GSH-dependent detoxification of RCS as well as of methylglyoxal and glyoxal; simultaneous maintenance of high GSH levels strongly favor detoxification (Yadav et al. 2005b, a; Davoine et al. 2005, 2006; Yin et al. 2017; Mano et al. 2019). The increased abundance of GSH and GSSG observed in apocarotenoid-accumulating roots supports the need for GSH-dependent detoxification of apocarotenoid-derived methylglyoxal and glyoxal via the glyoxalase pathway. However, we found little evidence for an induction of GLXI, GLXII and GLXIII at the transcript or protein level. None of the three known functional GLXI enzymes (At1g08110, At1g67280, At1g11840) were transcriptionally induced. This is further corroborated by a lack of increase in overall GLXI activity in *At12* and *At22* (Fig. 4). Nevertheless, two GLXI-like genes, *GlxI-like;4* and *GlxI-like;7*, were transcriptionally induced. However, the proteins encoded by these genes most probable do not have glyoxalase activity as the conserved GSH and metal ion binding sites present in the active GLXI proteins is missing (Schmitz et al. 2017, 2018). Similarly, among five *GLXIII* genes, only *At3g54600* but not *At3g02720* was induced transcriptionally upon apocarotenoid accumulation, the former coding for an isoform having only low activity on methylglyoxal in vitro whereas the latter coding for an isoform that has strong activity on methylglyoxal and glyoxal in vitro (Kwon et al. 2013). Lastly, it is noteworthy that only recently a methylglyoxal-specific GST was identified in *Synechocystis* which is essential for glyoxalase-based detoxification (Kammerscheit et al. 2020). Assuming that an *Arabidopsis* orthologue exists, the current understanding of glyoxalase pathway flux and its regulation might have to be revisited. Possible candidate genes might be found among the GSTs upregulated in *At12* and *At22* (Supplemental Table S1).

Interestingly, however, increased transcript and enzyme abundance of the relevant glyoxalases appears to be dispensable for apocarotenoid-derived methylglyoxal and glyoxal detoxification. This is supported by the fact that reaction velocity of GLXI, the rate-limiting enzyme of the pathway, is directly proportional to the concentration of GSH according to experimental data and mathematical modeling of its kinetics (Lages et al. 2012; Rabbani and Thornalley 2014; Nigro et al. 2017). Specifically, Lages et al. pointed out that the long-established non-enzymatic hemithioacetal formation, preceding the enzymatic GLXI reaction and its velocity depending on concentrations of methylgloyoxal or glyoxal and GSH, might limit substrate supply and reaction velocity of GLXI (Lages et al. 2012). In view of this, it is plausible that an increase in all reactants, GSH and methylglyoxal or glyoxal, in *At12* and *At22* per se, allows for increased hemithioacetal formation, increased substrate supply to GLXI and higher flux through the glyoxalase pathway without any increase in glyoxalase transcript and enzyme levels.

Additionally, there are other enzymes known to be capable of converting both methylglyoxal and glyoxal by non-GSH-dependent redox reactions which might complement the repertoire of detoxification pathways and can provide relief pressure on the GSH pool when cytotoxic compounds start depleting cellular GSH levels (Simpson et al. 2009; Yamauchi et al. 2011). Interestingly, among them are the enzymes AKR4C8, AKR4C9 and AtChlADR which are partially strongly induced in apocarotenoid-accumulating roots as we showed recently (Koschmieder et al. 2020). The contribution of GSH-independent enzymes has previously been debated under conditions of GSH depletion; however, the relative contribution of AKRs to methylglyoxal and glyoxal detoxification in bacteria, plants and animals remains under debate (Ko et al. 2005; Xu et al. 2006; Schumacher et al. 2018).

### Involvement of enzymes associated with photorespiration and the TCA cycle

The end products of the glyoxalase pathway for detoxification of mehylglyoxal and glyoxal are D-lactate and glycolate, respectively. Both represent central metabolites entering primary metabolism (Maurino and Engqvist 2015). D-lactate is converted by the mitochondrial D-LDH into pyruvate, which is an entry point into, inter alia, the TCA cycle and indirectly the methylerythritol phosphate pathway and thus carotenoid biosynthesis (Fig. 6; Zhao et al. 2013; Welchen et al. 2016). Similarly, glycolate is exclusively metabolized via glycolate oxidases, which are present in both photosynthetic and non-photosynthetic organs, into glyoxylate (Maurino and Engqvist 2015; Dellero et al. 2016; Modde et al. 2016). Supported by increased expression levels of malate synthase and malate dehydrogenase, respectively, we propose that glyoxylate is likely converted into oxaloacetate via malate. Oxaloacetate might be readily converted into pyruvate and feed IPP biosynthesis via the MEP pathway, which is partially supported by increased expression of phosphoenolpyruvate carboxykinases (Fig. 6).

Briefly, our data did not show any upregulation of D-LDH on the transcript level or increased levels of D-lactate or pyruvate (Fig. 5) at the interface between the glyoxalase pathway starting from methylglyoxal and the TCA cycle. However, primary metabolic pathways utilizing pyruvate have high reaction rates. It is thus conceivable that pyruvate is utilized at a sufficient rate to avoid accumulation, therefore compensating potential alterations at the D-lactate/pyruvate level, despite increased detoxification of apocarotenoid-derived methylglyoxal in *At12* and *At22*. Knocking out *D-LDH* in the *At12*/*At22* background and subsequent analysis of metabolite levels might reveal differences in glyoxalase pathway flux in future investigations.

In *At12* and *At22* roots, our data indicated upregulation of enzymes that in photosynthetic tissues are involved in the photorespiratory pathway. These activities most probably allow for sufficient metabolization of glyoxylate from glycolate generated from apocarotenoid-derived glyoxal by mechanisms discussed above (Fig. 6). Although glyoxylate amounts increased by twofold, those of glycolate remained constant in apocarotenoid-accumulating roots, again arguing for individual steps acting with high efficiency, thus not leading to increased intermediate abundance. Three enzymes that participate in the photorespiratory pathway in green tissues, namely, PLGG1, GOX1 and SGAT, were transcriptionally upregulated in roots of *At12* and *At22* (Table 3).

Partially high transcript levels as well as the presence of most proteins and their enzyme activities have been reported for photorespiratory genes in roots in addition to the presence of all “photorespiratory” metabolites (Nunes-Nesi et al. 2014; Engqvist et al. 2015; Schmitz et al. 2020). This suggests that enzymes associated with photorespiration in green tissues fulfill other physiological functions in heterotrophic organs such as roots. For instance, it was recently shown that isoforms of GOX serve different metabolic requirement in different tissues of *Ricinus*. While in autotrophic tissues, conversion of photorespiratory-generated glycolate into glyoxylate was considered as the main function, the activity of GOX isoforms in heterotrophic endosperm was assumed to likely warrant the availability of serine for the biosynthesis of folate (Schmitz et al. 2020). Nunes-Nesi et al. pointed out that 2-phosphoglycerate phosphatase, converting the Rubisco by-product 2-P-glycolate into glycolate, could not be detected in roots (Nunes-Nesi et al. 2014). In line with this, PGLP was not induced in *At12* and *At22*, suggesting that glycolate is formed from a different source than the oxygenase reaction of Rubisco, thus not requiring PGLP induction.

In summary, we provide evidence that carotenoid turnover is initiated by non-oxidative cleavage into the smallest units, methylglyoxal and glyoxal, followed by their subsequent detoxification into universal primary metabolites. Detoxification requires GSH which explains their higher levels in apocarotenoid-accumulating roots. Finally, detoxified methylglyoxal and glyoxal enter primary metabolism via the TCA cycle and enzyme activities associated with the photorespiratory pathway (Fig. 6). This might represent an ubiquitous recycling mechanism for carotenoid-derived carbon in plant tissues similar to the metabolism of vitamin E, another important cellular isoprenoid antioxidant, which involves regeneration of oxidized α-tocopherol into tocopherolquinone (Kobayashi and DellaPenna 2008). In view of the substantial carotenoid turnover in photosynthetic tissues which can be estimated to be about 14% of total leaf carotenoid amounts per day, substantial amounts of carotenoid-derived methylglyoxal and glyoxal can be expected (Simkin et al. 2003; Lätari et al. 2015). Maintenance of constant carotenoid levels thus requires the investment of energy in form of photosynthetically generated ATP; however, it requires minimum de novo carbon fixation. While in certain tissues, continuous carotenoid synthesis and breakdown might be important as these two processes allow homeostatic regulation of carotenoid levels and justify energy consumption, this might not be relevant in tissues with carotenoid-storage structures (Li and Yuan 2013). Importantly, in many of these structures, carotenoid oxidation is reduced, e.g., by sequestration into membranes as in daffodil flowers and thus reduces the need to recycle carotenoid carbon for their synthesis. In other tissues such as rice endosperm, absence of comparable sequestration structures results in continued carotenoid breakdown. High carotenoid pathway activity, e.g., by the endosperm-specific expression of carotenoid pathway enzymes in Golden Rice, might compensate oxidative degradation resulting in net carotenoid accumulation (Paine et al. 2005). However, with advanced rice endosperm development which is accompanied by apoptosis and thus an arrest of carotenoid biosynthetic flux, oxidation prevails and results in carotenoid losses (Paine et al. 2005; Schaub et al. 2017). While the multitude of carotenoid breakdown products complicates determining the progression of carotenoid degradation, exploitation of the universal carotenoid-derived metabolites and the enzymes involved in their continued metabolization identified in this work might help to associate genotypes with reduced carotenoid turnover with the molecular mechanisms involved and contribute to the stabilization of provitamin A in crops in future approaches.

## Methods

### Transcriptomics

RNA-seq analyses were performed on RNA isolated from roots of *Arabidopsis* wild-type ecotype Wassilewskija and two lines overexpressing *AtPSY* (line *At12* and line *At22*, Maass et al. 2009) as described previously (Koschmieder et al. 2020).

### GST activity measurements

GST activity in crude enzyme extracts from *Arabidopsis* roots was determined using a commercial GSH transferase assay kit (Sigma-Aldrich). Briefly, 50 µL of pre-cooled extraction buffer 1 [50 mM Tris–HCl, pH 7.5, 100 mM NaCl, 1 mM EDTA, 1 mM DTT and 0.5% (w/v) PVPP] was added to 50 mg of roots and ground in liquid nitrogen. Samples were resuspended thoroughly, kept on ice for 5 min and centrifuged at 21.000*g* for 15 min at 4 °C to obtain soluble crude enzyme preparation. Protein concentration was determined by Bradford assay and 35 µg total soluble protein was added to 200 µL GST assay (2 mM GSH, 1 mM CDNB in modified PBS). The increase in absorbance at 340 nm was monitored over 2 min every 30 s at room temperature and GST activity was calculated. For purified GSTs, 1 µg of enzyme was used.

### Purification of *Arabidopsis* GSTs

100 g *Arabidopsis* leaves (4–6 weeks old plants, long day conditions) were ground to powder in liquid nitrogen. Proteins were extracted with 150 ml pre-cooled extraction buffer 2 [50 mM Tris–HCl, pH 7.5, 100 mM NaCl, 1 mM EDTA, 1 mM DTT and 1% (w/v) PVPP] by sonication on ice. After 2 min centrifugation at 4.000*g*, the supernatant was filtered through one layer of Miracloth (20–25 µm) and the pellet was re-extracted with 100 ml pre-cooled extraction buffer 2. The combined protein extract was centrifuged for 60 min at 35.000*g* and 4 °C to remove membranes. Protein was precipitated at 80% ammonium sulfate saturation at 8 °C and stirring in a glass beaker for 1 h. The protein pellet obtained after centrifugation for 10 min at 30.000*g* and 4 °C was redissolved in 10 ml extraction buffer 2. Residual precipitate was removed by centrifugation at 21.000*g* for 5 min. The protein sample was desalted on PD10 columns (GE Healthcare) pre-equilibrated in extraction buffer 2. The protein sample was diluted 1:3 in extraction buffer 2 to 5–7 µg µL^−1^, and 400 µL GSH Sepharose 4B (GE Healthcare) was added and incubated for 4 h at 10 rpm overhead rotation and 8 °C. The resin was washed with 10 ml wash buffer [50 mM Tris–HCl/pH 7.5, 100 mM NaCl, 5% (v/v) glycerol, 1 mM EDTA, 1 mM DTT] twice. GSTs were eluted from the resin with 1 ml elution buffer [50 mM Tris–HCl/pH 8.0, 100 mM NaCl, 5% (v/v) glycerol, 1 mM DTT, 10 mM GSH] for 1 h at 8 °C and 10 rpm overhead rotation. GSTs were concentrated in Vivaspin hydrosart spin concentrators (30 kD MWCO, Merck). Protein concentration was determined by Bradford assay and GST purity was evaluated by 4–20% SDS-PAGE. GSTs were flash frozen in liquid nitrogen and stored in aliquots at −80 °C without significant loss of activity for several weeks.

### Non-enzymatic glutathionylation of apocarotenoids and 4-HNE

12 nmol apocarotenoids were dried from ethanolic stocks, redissolved in 10 µL DMSO and mixed with 190 µL assay buffer to yield the following final concentrations in 200 µL final volume: 60 µM apocarotenoid and 5–10 mM GSH in 50 mM Tris (pH 9.0) containing 5% (v/v) DMSO. The volatile apocarotenoids cyclocitral and ionone were directly added from their ethanolic stock to 10 µL DMSO; assays without GSH served as control. Assays were incubated for 2.5 h at 30 °C and extracted with 100 µL chloroform, 100 µL methanol and 10 µL α-tocopherylacetate (10 µg µL^−1^ in acetone) as internal standard. After centrifugation for 1 min at 17.000*g* and re-extraction with 100 µL chloroform, the combined hypophases were dried and dissolved in 40 µL chloroform/methanol (2:1, v/v). Assays with volatile apocarotenoids were extracted by partitioning against 40 µL chloroform/methanol (2:1, v/v) and the organic phase was directly transferred to an HPLC vial. The assay was re-extracted with 20 µL chloroform and the combined hypophase was mixed with methanol to obtain 70 µL chloroform/methanol (2:1, v/v) as final sample solvent. Remaining amounts of non-glutathionylated apocarotenoids were determined by HPLC, using a Shimadzu HPLC system with a C18 column (Hypersil Gold C18, 150 × 4.6 mm, 5 µm) and the mobile phases A: acetonitrile with 0.1% (v/v) formic acid and B: water with 0.1% (v/v) formic acid and a column temperature of 40 °C. 4–8 µL of sample was injected. Samples were separated with the following gradient at a flow rate of 1.5 ml min^−1^: hold 30% B for 3 min, from 30% B to 0% B within 7 min, hold 0% B for 3.5 min, back to 30% B in 30 s and re-equilibrate for 2.5 min.

For 4-hydroxynonenal (HNE), 24 nmol 4-hydroxynonenal was dried from ethanolic stock, dissolved in 4 µL DMSO, mixed with 190 µL PBS and supplemented with GSH to obtain the following final concentrations in 200 µL final volume: 120 µM HNE, 120 µM GSH in PBS (pH 7.4). Assays were incubated at room temperature for 15 min. For HPLC analysis, 200 µL acetonitrile with 0.1% (v/v) formic acid was added to stop the reaction at acidic pH. 4-HNE content was analyzed from 10 µL sample volume on a Shimadzu HPLC system with the above stationary and mobile phases. Samples were run at an isocratic flow rate of 1.25 ml min^−1^ with 50% B for 4 min. HNE was detected at its maximum absorption wavelength of 221 nm.

### Enzymatic glutathionylation of apocarotenoids with GSTs

24 nmol apocarotenoid stocks were prepared as described above, redissolved in 4 µL DMSO and mixed with 190 µL PBS to yield the following final concentrations in 200 µL final volume: 120 µM apocarotenoid and 120 µM GSH in PBS (pH 7.4) containing 2% (v/v) DMSO. 5 µg of purified *Arabidopsis* GST was added, and an equivalent volume of buffer served as non-enzymatic control. Assays were incubated for 4 h at 25 °C. Extraction and HPLC analysis to determine the remaining levels of non-glutathionylated apocarotenoids was carried out as described above.

### GlyI activity measurements

100 mg *Arabidopsis* root material ground to powder in liquid nitrogen was resuspended in 1 ml of pre-cooled extraction buffer 3 [50 mM HEPES/pH 7.5, 100 mM NaCl, 5 mM MgCl_2_, 10% (v/v) glycerol, 0.25% (w/v) PVPP]. The protein concentration was determined from the supernatant obtained after 15 min at 4 °C and 100.000 g by Bradford assay. The combined specific activity of GlyI enzymes was determined according to Arai et al. (2014) with slight modifications. The assay buffer (50 mM sodium phosphate buffer/pH 6.6, 2 mM methylglyoxal, 2 mM reduced GSH) was pre-incubated for 10 min at 37 °C to allow for non-enzymatic formation of hemithioacetal as GlyI substrate. 5 µg total protein in 10 µL buffer was added to 490 µL assay buffer, incubated at room temperature and the increase in absorption at 240 nm was monitored for 10 min every 5 min. GlyI activity was determined based on the molar extinction coefficient of 3.37 mM^−1^ cm^−1^ at 240 nm for the formed S-lactoylglutathione.

### Metabolic profiling

Extraction and analysis by gas chromatography coupled with mass spectrometry was performed using the same equipment setup and exactly the same protocol as described in Lisec et al. (2006). Briefly, frozen ground material was homogenized in 300 μL of methanol at 70 °C for 15 min and 200 μL of chloroform, followed by addition of 300 μL of water. The polar fraction was dried under vacuum, and the residue was derivatized for 120 min at 37 °C (in 40 μl of 20 mg ml^−1^ methoxyamine hydrochloride in pyridine), followed by a 30 min treatment at 37 °C with 70 μl of *N*-methyl-*N*-trimethylsilyltrifluoracetamide (MSTFA). An autosampler multi-purpose system (Gerstel GmbH & Co.KG, Mülheim an der Ruhr, Germany) was used to inject the samples to a chromatograph coupled to a time-of-flight mass spectrometer (GC–MS) system (Leco Pegasus HT TOF-MS, LECO Corporation, St. Joseph, MI, USA). Helium was used as carrier gas at a constant flow rate of 2 ml s^−1^ and gas chromatography was performed on a 30 m DB-35 column. The injection temperature was 230 °C and the transfer line and ion source were set to 250 °C. The initial temperature of the oven (85 °C) increased at a rate of 15 °C min^−1^ up to a final temperature of 360 °C. After a solvent delay of 180 s mass spectra were recorded at 20 scans s^−1^ with *m*/*z* 70–600 scanning range. Chromatograms and mass spectra were evaluated by using Chroma TOF 4.5 (LECO) and TagFinder 4.2 software.

### LC–MS analysis

LC–MS analyses were performed as described previously (Koutouan et al. 2018), except that freeze-dried root powder was extracted with 30 µL mg^−1^ of methanol containing 5 μg mL^−1^ of chloramphenicol as an internal standard. Molecular ions [M + H]^+^ corresponding to putative adducts of the apocarotenoids detected in the *At12* and *At22* lines (Koschmieder et al. 2020) were searched based on their predicted molecular formula using the Xcalibur software (Thermo Fischer). Detection of other GSH adducts described previously (Davoine et al. 2005) was performed as a validation of this approach.

## Supplementary Information

Below is the link to the electronic supplementary material.

Supplementary file1 (PDF 854 KB)

## Data Availability

Not applicable.
