# Supplementary material for: Color recycling: metabolization of apocarotenoid degradation products suggests carbon regeneration via primary metabolic pathways
Source: Plant Cell Rep. 2022 Jan 22;41(4):961–77. doi: 10.1007/s00299-022-02831-8 (PMC9035014; doi:10.1007/s00299-022-02831-8)
Supplement: Supplementary file 1 — Supplementary file1 (PDF 854 KB) [file 299_2022_2831_MOESM1_ESM.pdf]

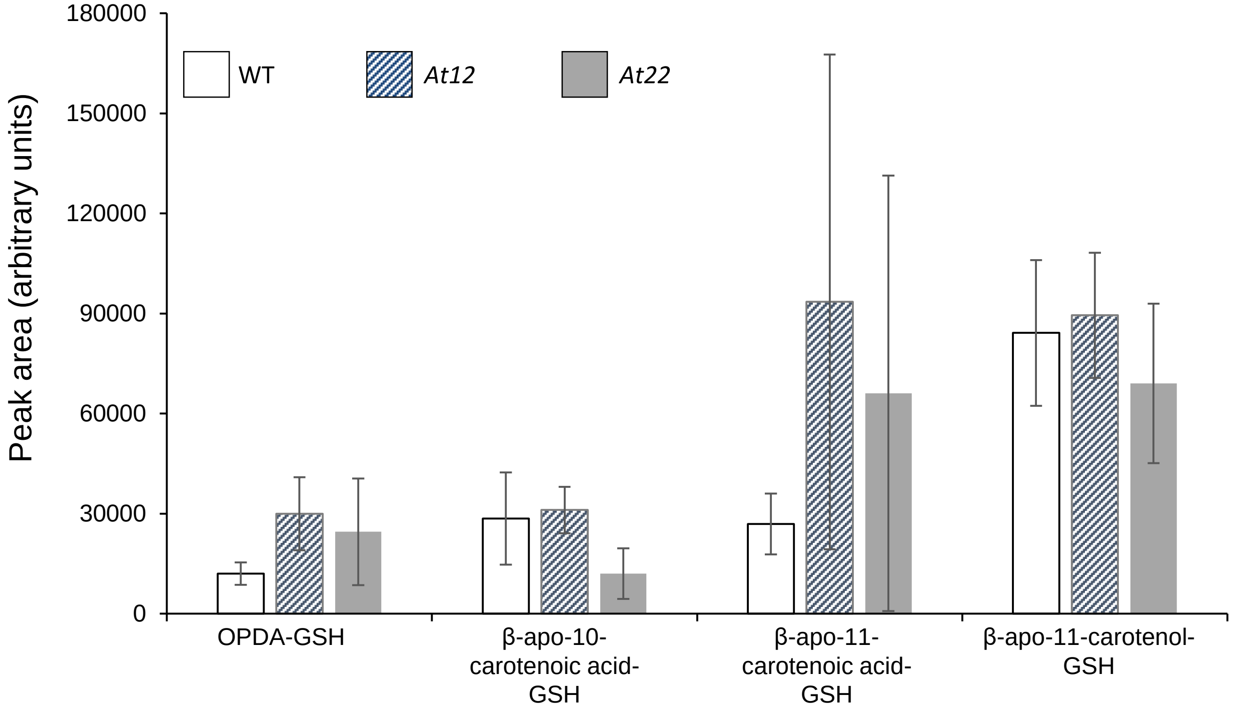

Figure S1: Detection of glutathione adducts in Arabidopsis roots

OPDA-GSH and putative apocarotenoid glutathione adducts were detected in three biological replicates of roots from hydroponically grown wild-type and *AtPSY*-overexpressing Arabidopsis lines *At12* and *At22*.

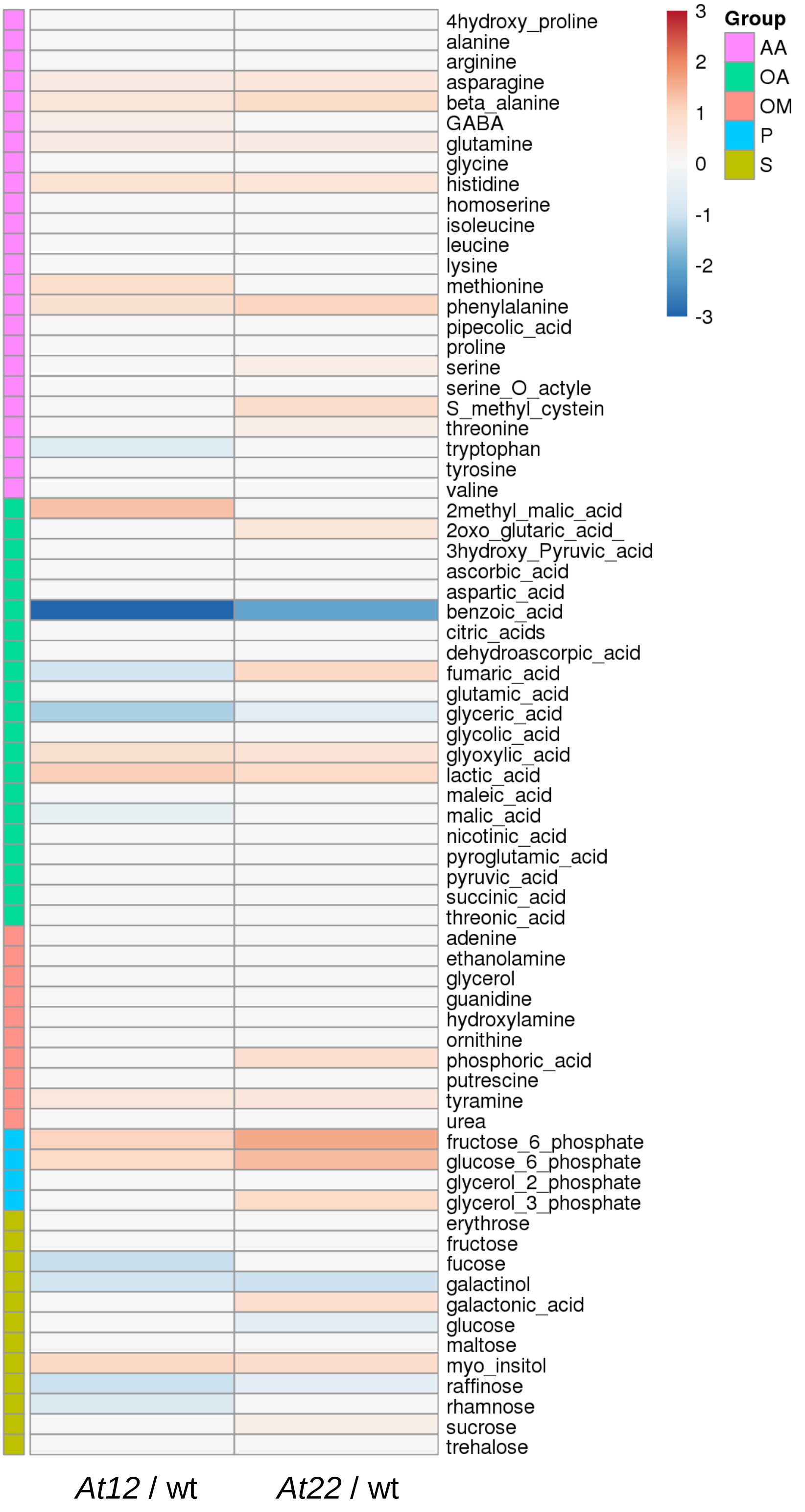

Figure S2: Differential metabolic profiling of wild-type and carotenoid-accumulating Arabidopsis roots

Metabolites were analyzed from roots of hydroponically grown wild-typ and *AtPSY*-overexpressing Arabidopsis lines *At12* and *At22*. Log2 of significant metabolite fold changes for indicated pairwise comparisons are given by shades of red or blue colors according to the scale bar. Metabolites were grouped according to their chemical or functional family as amino acids and derivatives (AA), organic acids (OA), other metabolites (OM), organic phosphates (P), and sugars and derivatives (S). Data represent mean values of three biological replicates for each line. Statistical analysis was performed using Tukey's honest significant difference method followed by a false discovery rate (FDR) correction, with FDR < 0.05. For FDR ≥ 0.05, Log2 fold changes were set to 0.

GST gene	fold induction for At22 / At12	primary substrate
GSTU subfamily		
At2g29450 (AtGSTU5)	4 / < 2	4-hydroxynonenal
At3g09270 (AtGSTU8)	6 / 11	?
At1g59700 (AtGSTU16)	3 / 6	?
Atg78380 (AtGSTU19)	2 / 3	acrolein
At1g78370 (AtGSTU20)	2 / 3	?
At1g17170 (AtGSTU24)	3 / 3	acrolein
At1g17190 (AtGSTU26)	7 / 3	?
GSTF subfamily		
At1g02930 (AtGSTF6)	3 / 3	n.a.
At1g02920 (AtGSTF7)	3 / 3	n.a.
At2g30860 (AtGSTF9)	2 / 2	n.a.
At3g03190 (AtGSTF11)	6 / 3	n.a.

Table S1: Induced GST transcript levels in Arabidopsis roots in response to β-carotene and β-apocarotenoid accumulation
RNA-Seq-determined expression changes in roots of *AtPSY*-overexpressing lines *At22* and *At12* relative to wild-type roots are shown for GST genes with at least 2-fold induction. Known substrates are given based on *in vitro* assays with purified GSTUs reported by Mano *et al.* (2019) and are not available (n.a.) for GSTFs.
